# Induction of STK11-dependent cytoprotective autophagy in breast cancer cells upon honokiol treatment

**DOI:** 10.1038/s41420-020-00315-w

**Published:** 2020-09-06

**Authors:** Nethaji Muniraj, Sumit Siddharth, Marey Shriver, Arumugam Nagalingam, Sheetal Parida, Juhyung Woo, Justin Elsey, Kathleen Gabrielson, Edward Gabrielson, Jack L. Arbiser, Neeraj K. Saxena, Dipali Sharma

**Affiliations:** 1 Department of Oncology, Johns Hopkins University School of Medicine and the Sidney Kimmel Comprehensive Cancer Center at Johns Hopkins, Baltimore, MD 21231 USA; 2 Department of Pathology, Johns Hopkins University School of Medicine and the Sidney Kimmel Comprehensive Cancer Center at Johns Hopkins, Baltimore, MD 21231 USA; 3 Department of Dermatology, Emory School of Medicine, Atlanta Veterans Administration Medical Center, Atlanta, GA 30322 USA; 4 Molecular and Comparative Pathobiology, Johns Hopkins University School of Medicine and the Sidney Kimmel Comprehensive Cancer Center at Johns Hopkins, Baltimore, MD 21231 USA; 5 Early Detection Research Group, National Cancer Institute, Rockville, MD USA

**Keywords:** Breast cancer, Tumour-suppressor proteins

## Abstract

Cancer cells hijack autophagy pathway to evade anti-cancer therapeutics. Many molecular signaling pathways associated with drug-resistance converge on autophagy induction. Honokiol (HNK), a natural phenolic compound purified from *Magnolia grandiflora*, has recently been shown to impede breast tumorigenesis and, in the present study, we investigated whether breast cancer cells evoke autophagy to modulate therapeutic efficacy and functional networks of HNK. Indeed, breast cancer cells exhibit increased autophagosomes-accumulation, MAP1LC3B-II/LC3B-II-conversion, expression of ATG proteins as well as elevated fusion of autophagosomes and lysosomes upon HNK treatment. Breast cancer cells treated with HNK demonstrate significant growth inhibition and apoptotic induction, and these biological processes are blunted by macroautophagy/autophagy. Consequently, inhibiting autophagosome formation, abrogating autophagosome-lysosome fusion or genetic-knockout of *BECN1* and *ATG7* effectively increase HNK-mediated apoptotic induction and growth inhibition. Next, we explored the functional impact of tumor suppressor STK11 in autophagy induction in HNK-treated cells. STK11-silencing abrogates LC3B-II-conversion, and blocks autophagosome/lysosome fusion and lysosomal activity as illustrated by LC3B-Rab7 co-staining and DQ-BSA assay. Our results exemplify the cytoprotective nature of autophagy invoked in HNK-treated breast cancer cells and put forth the notion that a combined strategy of autophagy inhibition with HNK would be more effective. Indeed, HNK and chloroquine (CQ) show synergistic inhibition of breast cancer cells and HNK-CQ combination treatment effectively inhibits breast tumorigenesis and metastatic progression. Tumor-dissociated cells from HNK-CQ treated tumors exhibit abrogated invasion and migration potential. Together, these results implicate that breast cancer cells undergo cytoprotective autophagy to circumvent HNK and a combined treatment with HNK and CQ can be a promising therapeutic strategy for breast cancer.

## Introduction

Despite tremendous progress in encouraging regular screenings, identifying breast lesions at earlier stages and availability of multiple combination therapeutic strategies, breast cancer remains the second leading cause of cancer-related mortality in women^1,2^. Major hurdles include poor de novo response to therapies; development of acquired resistance leading to recurrent disease and metastasis; and non-compliance in patients owing to poor tolerance of drug-related side effects. Also, cancer cells prove to be evolving targets as they develop reliance on additional signaling networks when one signaling node is targeted. While we have multiple successful single-target drugs, we need to develop multi-target strategies as targeting multiple oncogenic signaling pathways simultaneously may prove more useful than targeting single nodes. Hence, important requirements for an ideal breast cancer therapy or prevention strategy are the ability to inhibit multiple subtypes of breast cancer, simultaneously impede multiple key kinases, few/no side effects and oral availability.

An interesting approach adopted by cancer cells to evade any therapy is the onset of cytoprotective autophagic process. Macroautophagy (referred as autophagy hereafter) is generally utilized by normal cells to avoid accumulation of damaged proteins or organelles, reduce ER stress and lower reactive oxygen species (ROS) production but cancer cells hijack this process to survive cancer therapy^3^. Lysosomal machinery degrades damaged cytoplasmic components of the cells and yield macromolecular constituents that can be recycled to maintain cellular metabolism^4,5^. Multiple steps of autophagy such as formation of induction-complex, vesicle nucleation, autophagosome formation, autolysosome formation and finally, the disintegration of the cargo^6,7^, are regulated by various autophagy-related (ATG) proteins^6,8^. Multiple pathways including PIK3CA/AKT and AMPK/TSC1/2 signaling networks regulate autophagy^9^. Autophagic induction in response to hypoxia, DNA-damage or chemotherapy leads to the development of chemoresistance^10–12^. In light of the importance of autophagic induction in evading/reducing drug efficacy, it is imperative to investigate whether breast cancer cells induce autophagy in response to a proposed/established cancer therapy.

Bioactive compounds or nutraceuticals, natural constituents of plants and certain foods, have shown efficacy as anti-inflammatory, anti-depressants, anti-microbial agents as well as anti-cancer agents^13^. Bioactive compound purified from the seed cones and bark extracts of *Magnolia grandiflora*, Honokiol (HNK), is a small phenolic compound that has been used in traditional medicine and has successfully crossed the bridge to modern medical research^14^. HNK effectively inhibits growth and induce cell cycle arrest and apoptosis in multiple subtypes of breast cancer^15,16^. Cell cycle arrest mediated by HNK is a coordinated action achieved by inhibition of cyclin D1, cyclin E1, cyclin dependent kinase 2, cyclin dependent kinase 4, cMYC and RB along with abrogation of CSK/EGFR signaling^17^. Acquiring increased invasion and migration potential are key aspects of tumor progression and HNK inhibits migration of breast cancer cells via inhibiting nitric oxide and prostaglandin-endoperoxidase synthase 2/cyclooxygenase-2^18^. Our group investigated the signaling networks underlying anti-breast cancer potential of HNK and showed the involvement of AMPK and LKB1^19^. Epithelial-to-mesenchymal transition (EMT) is another critical step in metastatic progression, and HNK treatment effectively inhibits EMT in breast cancer cells by abrogating activation of STAT3 which leads to inhibition of ZEB1 and upregulation of cadherin 1^20^. Delving further into the mechanistic aspects of HNK function, our group showed that HNK effectively inhibits oncogenic signaling of adipocytokine leptin by activating LKB1-miR34a axis and inhibiting WNT1-MTA1-βcatenin axis^21,22^. Intriguingly, HNK successfully inhibits pluripotency factors POU5F1, Nanog and SOX2 and abrogates breast cancer stem-like phenotype^23^. These important studies not only established the effectiveness of HNK in inhibiting breast cancer growth, stemness and metastatic progression but also put forth HNK as a multikinase inhibitor with oral bioavailability^21–23^.

In this study, we investigated whether breast cancer cells initiate an autophagic response to HNK treatment. We report a stimulation of autophagic flux in breast cancer cells upon HNK treatment and suppressing autophagy with inhibition of autophagosome formation using 3-methyladenine, blockade of autophagosome-lysosome fusion with bafilomycin A_1_ (Baf), or CRISPR/Cas9-mediated knockout of *beclin 1* (*BECN1*) and *autophagy related 7* (*ATG7*) effectively potentiates HNK induced growth inhibition and apoptotic induction. We also show that tumor suppressor STK11 is important for initiating autophagy in HNK-treated breast cancer cells as STK11 silencing abrogates LC3B-II puncta, autophagosome/lysosome fusion and lysosomal activity. We further investigated the effect of combining an autophagy inhibitor with HNK on breast tumorigenesis and show that a combination regimen of HNK and chloroquine is more effective in reducing breast tumor growth as well as lung metastasis.

## Results

### Breast cancer cells exhibit increased accumulation of intracellular autophagosomes and autophagy markers upon HNK treatment

**Fig. 1:**
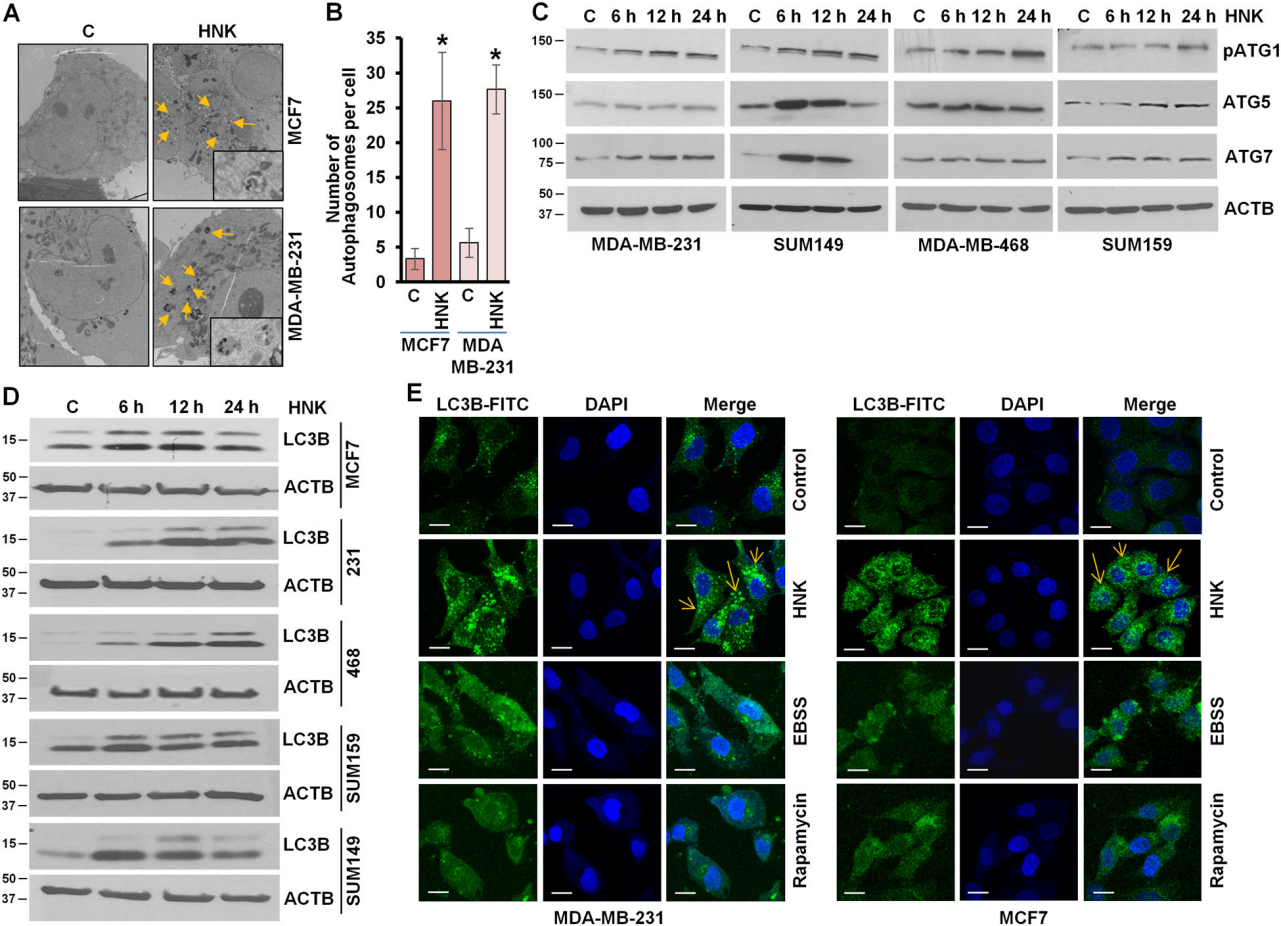
HNK induces autophagosome accumulation, LC3B conversion, and other autophagy related proteins. **a** MCF7 and MDA-MB-231 cells were treated with 5 µM HNK for 24 h and visualized under an electron microscope. Representative pictures are shown with approximately ×7400 magnification (×50000 in the highlighted area). **b** Double-membrane autophagosomes were counted in randomly selected ~100 cells in random fields. Number of autophagosomes per cell is shown in bar graphs. *P < 0.05, compared with vehicle-treated controls (**c**) in MCF7 and MDA-MB-231 cells. **c** Immunoblot analysis of pATG1, ATG5, ATG7, and BECN1 in breast cancer cells treated with 5 µM HNK for indicated time intervals. ACTB was used as a loading control. **d** Breast cancer cells were treated with 5 µM HNK for indicated time intervals and total cell lysates were immunoblotted for LC3B expression. ACTB was used as a loading control. **e** Breast cancer cells were treated with 5 µM HNK or 200 nM rapamycin or Earle’s balanced salt solution (EBSS) for 24 h and subjected to immunocytochemistry using LC3B antibody. Scale bars: 20 µm. Representative immunofluorescence images are shown.

We examined whether breast cancer cells invoke autophagic response upon treatment with HNK. Ultrastructural changes in breast cancer cells were observed using transmission electron microscopy (TEM). The TEM studies uncovered that breast cancer cells treated with 5 μM HNK for 24 h attained significantly higher number (5–6 fold increase) of autophagic vacuoles as compared to control cells (Fig. 1a, b). All the steps of autophagy progression are under tight regulation of ATG proteins, such as ATG1, ATG5, ATG7, and BECN1^7^. Multiple breast cancer cells, MDA-MB-231, SUM149, MDA-MB-468, and SUM159 showed increased expression of pATG1, ATG5, ATG7 and BECN1 proteins in a temporal manner in response to HNK treatment (Fig. 1c, Supplementary Fig. 1). An important node in autophagy, MAP1LC3B/LC3B (microtubule associated protein 1 light chain 3 alpha) is cleaved by ATG4 to generate the cytoplasmic-form LC3B-1 (18 kDa) that gets converted to phagophore-associated LC3B-II (16 kDa) form upon conjugation with the lipid phosphatidylethanolamine^24^. We examined conversion of LC3B-I to LC3B-II in HNK-treated breast cancer cells, and found a time-dependent increase in LC3B-II (LC3B) in MCF7, MDA-MB-231, MDA-MB-468, SUM159, and SUM149 cells (Fig. 1d). Spatial redistribution of LC3B from cytosol to autophagosomes accompanies the formation of autophagosomes and can be detected as LC3B puncta with confocal microscopy. HNK-treated breast cancer cells exhibited a marked increase in LC3B puncta formation in comparison to control cells which exhibited a diffuse green fluorescence. Breast cancer cells treated with rapamycin and EBSS, known stimulators of autophagy, also showed increased presence of LC3B puncta (Fig. 1e). These results suggest that breast cancer cells accumulate increased number of autophagosomes and redistribute LC3B in response to HNK.

### Increased fusion of autophagosomes and lysosomes in HNK-treated breast cancer cells

**Fig. 2:**
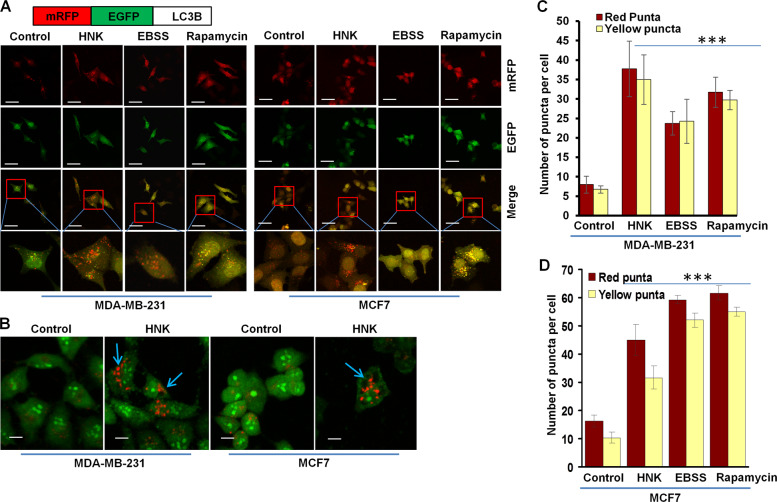
HNK augments autophagy in breast cancer cells. **a** Schematic diagram of the tfLC3 plasmid (upper panel). MDA-MB-231 and MCF7 cells were transfected with tfLC3 followed by treatment with 5 µM HNK or 200 nM rapamycin or Earle’s balanced salt solution (EBSS) for 24 h. Rapamycin and EBSS were used as positive controls for autophagic induction. Representative fluorescent images are shown. Scale bar: 10 µm. **b** MDA-MB-231 and MCF7 cells were treated with 5 µM HNK for 24 h followed by acridine orange staining. Representative images of MDA-MB-231 and MCF7 cells are shown. Scale bar: 15 µm. **c, d** Bar graphs show number of red and yellow puncta per cell in MDA-MB-231 and MCF7 cells transfected with tfLC3 and treated with 5 µM HNK or 200 nM rapamycin or EBSS for 24 h. ***P < 0.0005, compared with control.

**Fig. 3:**
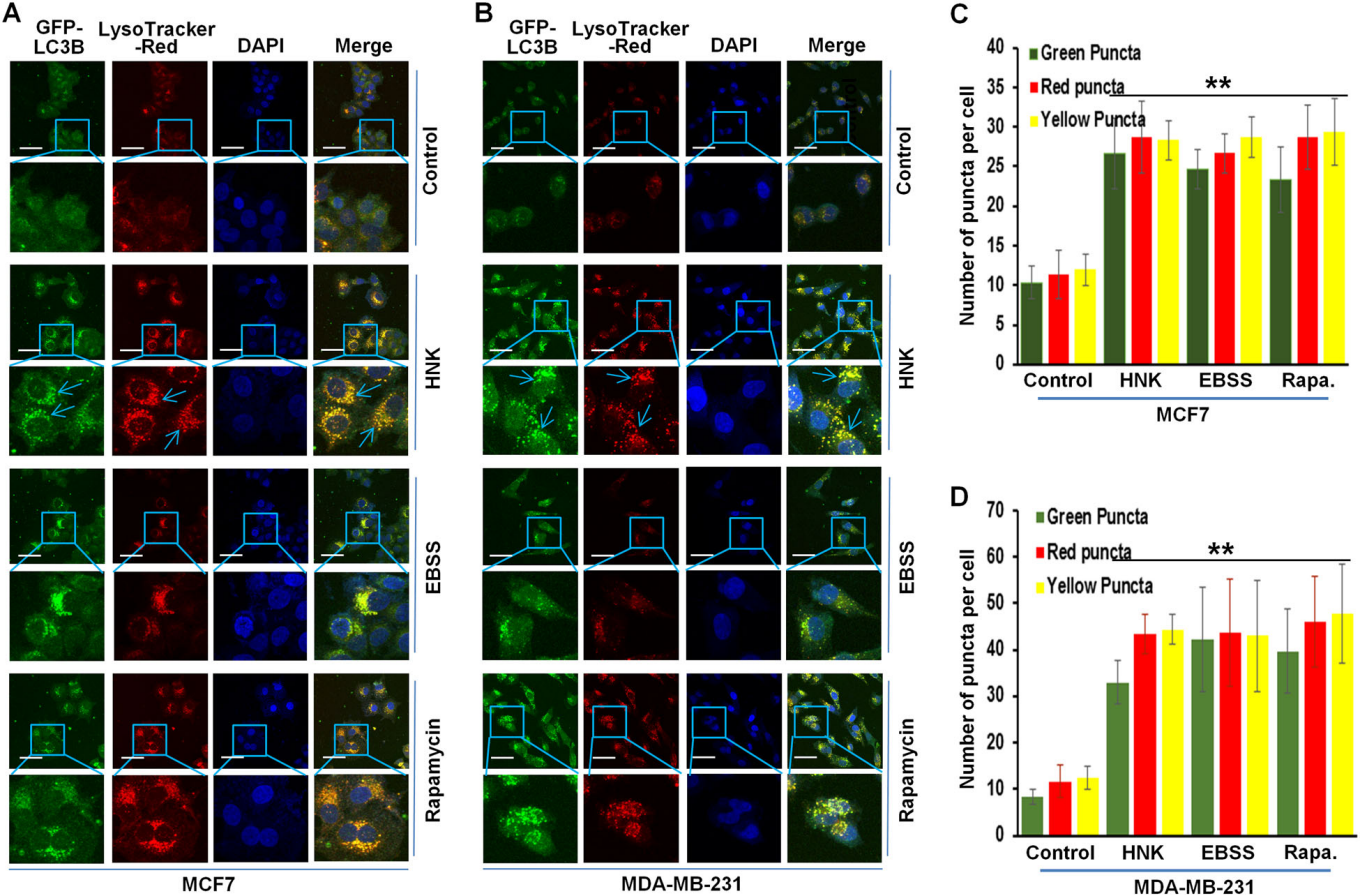
Increased fusion of autophagosomes and lysosomes in breast cancer cells treated with HNK. **a, b** MCF7 and MDA-MB-231 cells were transfected with GFP-LC3B followed by treatment with 5 µM HNK or 200 nM rapamycin or EBSS for 24 h and staining with LysoTracker-Red. Cells were fixed and subjected to confocal microscopy. Representative fluorescent images are shown. Scale bar: 10 µm. **c, d** Bar graphs show green, red and yellow puncta per cell in MDA-MB-231 and MCF7 cells. **P < 0.005, compared with control.

Increased levels of LC3B expression and puncta can be indicative of either an increased synthesis or a decreased turnover of autophagosomes owing to a delay in trafficking to the lysosomes and fusion with lysosomes^25^. To query these important notions, a plasmid encoding membrane-localized red fluorescent protein (mRFP)-EGFP-LC3B (tandem fluorescent-tagged LC3B [tfLC3B]) resulting in both green and red fluorescence^26^ was transfected in breast cancer cells. Autophagosomes (GFP-positive and RFP-positive, merged as yellow puncta) and autolysosomes (GFP-negative and RFP-positive, merged as red puncta) can be observed in cells transfected with tfLC3B as EGFP fluorescence gets quenched in acidic compartments whereas mRFP remains stable in low-pH environment. Autophagy induction increases both yellow and red puncta formation. Breast cancer cells exhibited an increase in both yellow (autophagosomes) and red (lysosomes) puncta upon HNK treatment indicating an increase in autophagic flux. EBSS and rapamycin treatment also increased yellow and red puncta in breast cancer cells (Fig. 2a, c, d). A characteristic feature of autolysosomes is acidic pH which can be evaluated with acridine orange staining. Acridine orange is a nucleic acid dye that becomes protonated and sequestered in acidic compartments like lysosomes and emits red fluorescence when excited by blue light. Nucleus and cytoplasm of breast cancer cells stained with acridine orange showed green fluorescence while a red and orange fluorescence marked acidic compartments. HNK treatment induced the acidic compartments visualized as red/orange puncta (Fig. 2b). Cells can be costained for GFP-LC3B (green puncta, localized on autophagosomes) and LysoTracker Red (red puncta, an acidic pH marker for lysosomes) to investigate the fusion of autophagosomes and lysosomes. Breast cancer cells transfected with GFP-LC3B were stained with LysoTracker Red; confocal microscopy revealed increased overlap between GFP-LC3B and LysoTracker Red puncta (observed as yellow puncta) in HNK-treated cells as compared to control cells demonstrating an increased autophagosomes-lysosomes fusion. MCF7 and MDA-MB-231 cells treated with EBSS and rapamycin also exhibited increased autophagosomes-lysosomes fusion (yellow puncta) in breast cancer cells (Fig. 3a–d). Collectively, these data provide strong evidence that breast cancer cells exhibit an increase in autophagy upon HNK treatment.

### Breast cancer cells initiate cytoprotective autophagy upon HNK treatment to reduce HNK-mediated growth inhibition and apoptotic induction

**Fig. 4:**
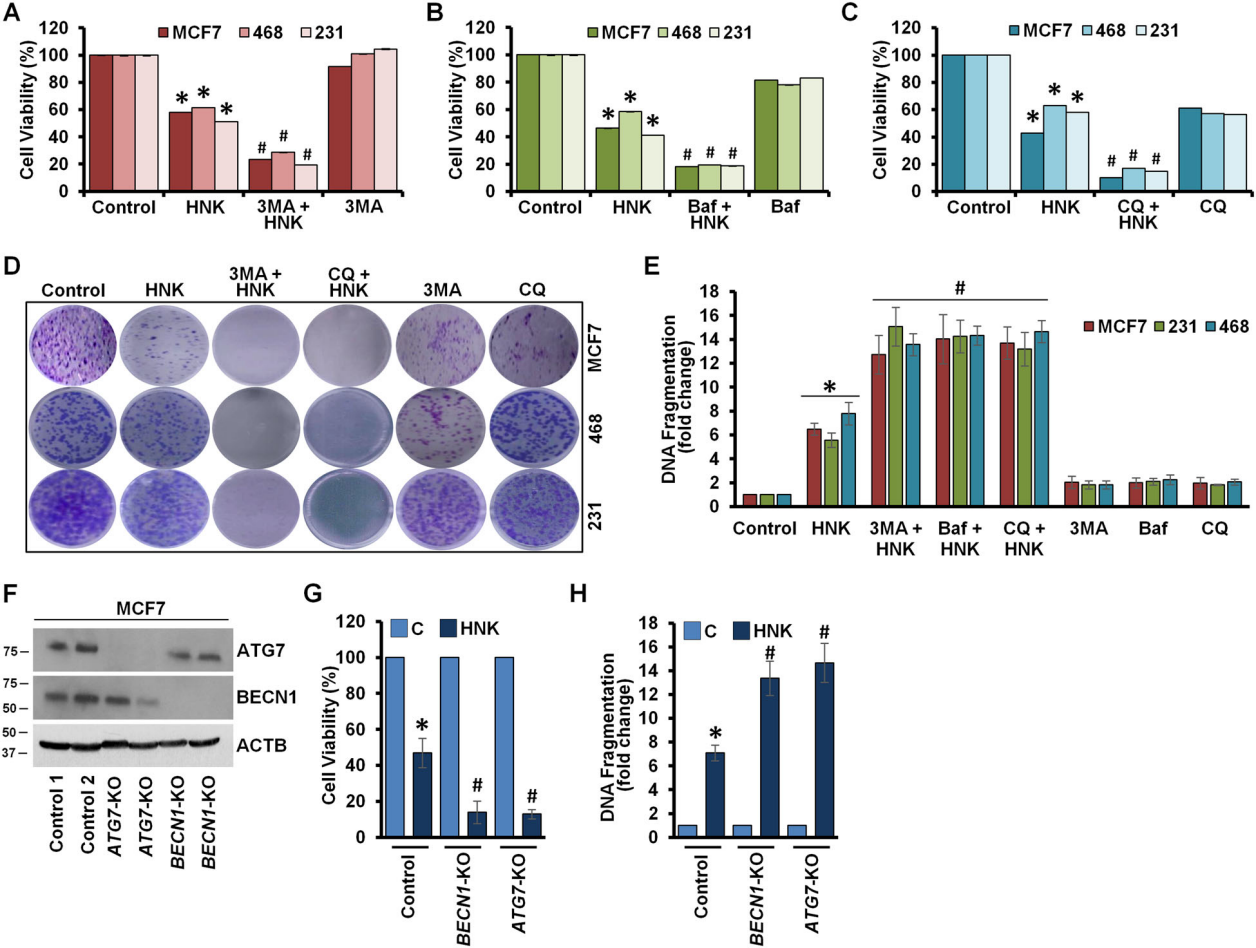
Inhibition of autophagy increases HNK-mediated reduction in cell-survival and apoptosis-induction in breast cancer cells. **a**–**c** MCF7, MDA-MB-468 and MDA-MB-231 cells were treated with 5 µM HNK alone or in combination with 4 mM 3-methyladenine (3MA) or 200 nM bafilomycin A_1_ (Baf) or 25 μM chloroquine (CQ) for 24 h as indicated and subjected to MTT assay. *P < 0.05, compared with control; ^#^*P* < 0.05, compared with HNK. **d** MCF7, MDA-MB-468, and MDA-MB-231 cells were treated with 5 µM HNK, 4 mM 3MA or 25 μM CQ alone or in combination as indicated for 24 h and subjected to clonogenicity assay. Representative pictures are shown for each treatment. **e** Breast cancer cells were treated with 5 µM HNK, 4 mM 3MA, 200 nM Baf, 25 μM CQ alone or in combination as indicated for 24 h and subjected to DNA-fragmentation assay. *P < 0.05, compared with control; ^#^*P* < 0.05, compared with HNK. **f** CRISPR/Cas9 was used to knockout *BECN1* and *ATG7* in MCF7 cells and total cell lysates were immunoblotted for BECN1 and ATG7. ACTB was used as loading control. **g** Cell viability of control, *BECN1-KO and AT****G****7-KO* MCF7 cells was examined using MTT assay after treatment with 5 µM HNK for 24 h. *P < 0.05, compared with vehicle control; ^#^*P* < 0.05, compared with HNK-treated cells. **h** Vector-control, *BECN1-KO and ATG7-KO* MCF7 cells were treated with 5 µM HNK for 24 h and subjected to DNA-fragmentation assay. *P < 0.05, compared with vehicle control; ^#^*P* < 0.05, compared with HNK-treated cells.

Induction of autophagy can have different functional impacts on the cells. “Cytostatic autophagy” leads to growth inhibition and senescence whereas a “nonprotective autophagy” does not impact therapeutic sensitivity of the cells. Autophagy is considered “cytoprotective” when it bestows therapeutic resistance and its blockade aids in cell death, contrastingly, a “cytotoxic autophagy” promotes apoptosis and increases drug efficacy^27–29^. To examine the functional impact of autophagy triggered in response to HNK, we blocked autophagy in HNK-treated breast cancer cells by inhibiting autophagosome formation using 3-methyladenine (3MA), a phosphatidylinositol 3-kinase (PtdIns3K/Vps34) inhibitor^30^, or impeding autophagosome-lysosome fusion using Baf, a specific vacuolar type H^+^-ATPase inhibitor^31^ or increasing lysosomal pH using chloroquine, a weak base. Interestingly, we found that autophagy inhibition with 3MA, Baf or CQ in HNK-treated breast cancer cells further decreased cell survival (10–25%) in comparison to HNK treatment alone (40–60%) (Fig. 4a–c, Supplementary Fig. 2A, B). Evaluation of clonogenic potential showed reduced clonogenicity in breast cancer cells treated with a combination of 3MA, Baf or CQ with HNK compared to HNK treatment alone (Fig. 4d). HNK treatment induced significant apoptotic cell death as observed in DNA-fragmentation assay, interestingly; autophagy inhibition with 3MA, Baf or CQ in combination with HNK further enhanced its impact (6–7-fold vs. 13–15-fold) (Fig. 4e). BECN1 and ATG7 are important proteins mediating the multistep autophagic process^32^. We knocked out *BECN1* and *ATG7* using CRISPR/Cas9 technology in MCF7 cells as a genetic intervention. MCF7 cells knocked out for *ATG7* showed intact BECN1 and cells knocked out for *BECN1* showed intact ATG7 in both clones exhibiting the specificity (Fig. 4f). HNK-mediated reduction in cell survival was further enhanced in *BECN1*-KO and *ATG7*-KO MCF7 cells compared to control cells (Fig. 4g). Also, *BECN1*-KO and *ATG7*-KO MCF7 cells exhibited significantly higher apoptotic cell death upon HNK treatment in comparison to HNK-treated control cells (Fig. 4h). Taken together, these results show that sensitivity to HNK-mediated growth inhibition and apoptotic induction is significantly improved by impairing autophagy indicating that breast cancer cells initiate a cytoprotective autophagy to evade HNK.

### STK11 silencing attenuates cytoprotective autophagy in HNK-treated breast cancer cells

**Fig. 5:**
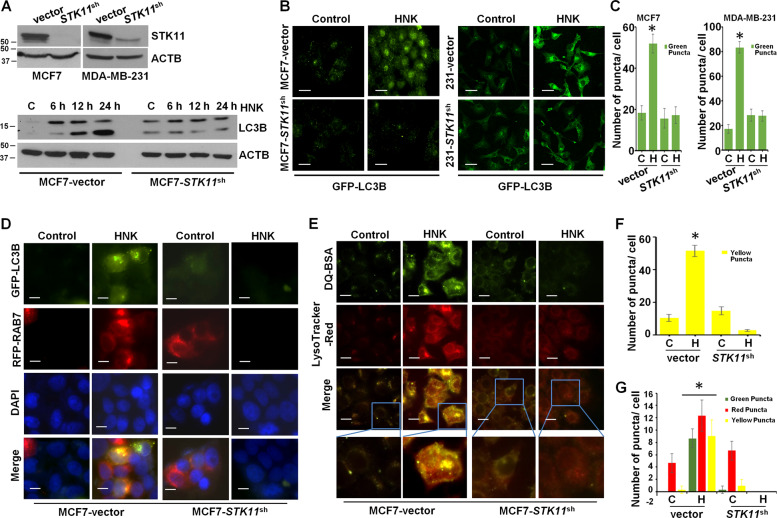
STK11 silencing attenuates HNK-mediated cytoprotective autophagy in breast cancer cells. **a** Total protein lysates of MCF7 and MDA-MB-231 cells transfected with *STK11*^shRNA^ and control vector-pLKO.1 (vector) were immunoblotted for the expression of STK11. MCF7-pLKO.1 (vector) and *STK11*^shRNA^ cells were treated with 5 µM HNK for indicated times and immunoblotted for the expression of LC3B. **b, c** MCF7-pLKO.1 (vector), MCF7-*STK11*^shRNA^, MDA-MB-231-pLKO.1 (vector) and MDA-MB-231-*STK11*^shRNA^ were treated with 5 µM HNK for 24 h and subjected to immunocytochemistry using LC3B antibody. Scale bars: 20 µm. Representative immunofluorescence images are shown. Bar graphs show number of LC3B puncta per cell. *P < 0.05. **d, f** MCF7-pLKO.1 control (vector) and *STK11*^shRNA^ cells were co-transfected with GFP-LC3B and RFP-RAB7 followed by treatment with 5 µM HNK for 24 h. Fixed cells were subjected to immunofluoroscent microscopy. Representative fluorescent images are shown. Scale bars: 10 µM. Bar graph shows number of red, green and yellow puncta per cell. *P < 0.05. **e, g** MCF7-pLKO.1 (vector) and *STK11*^shRNA^ cells were incubated with 10 µg/ml DQ-BSA for 2 h followed by treatment with 5 µM HNK for 24 h. Cells were fixed and stained with LysoTracker-Red followed by immunofluoroscent imaging. Representative images are shown. Scale bars: 10 µM. Bar graph shows number of yellow puncta per cell. *P < 0.05.

STK11, tumor suppressor and an upstream kinase, has been shown to be involved in promoting autophagy in response to nutrient-deprivation, therapeutics and other environmental cues^33–36^. HNK induced expression of STK11 and pAMPK, its downstream kinase target (Supplementary Fig. 3A). Cells treated with HNK also exhibited inhibition of p70 kDa ribosomal protein S6 kinase 1 (p70S6K1 or pS6K) and the eukaryotic translation initiation factor 4E (elF4E)-binding protein (4EBP1), two downstream effectors of mTOR indicative of mTOR activity (Supplementary Fig. 3B). We raised the question whether STK11 plays any role in autophagy induction in breast cancer cells treated with HNK. We selected stable pools of MCF7 and MDA-MB-231 cells with STK11 depletion utilizing *STK11*^shRNA^ lentiviruses and puromycin selection and confirmed the knockdown of STK11 protein in immunoblot analyses (Fig. 5a). We observed that MCF7 cells infected with *STK11* shRNA showed abrogation of LC3B conversion while MCF7 cells infected with vector exhibited increased levels of LC3B conversion upon HNK treatment (Fig. 5a). Confocal microscopy detected increased LC3B puncta formation in MCF7-vector and MDA-MB-231-vector-control cells treated with HNK while MCF7-*STK11*^shRNA^ and MDA-MB-231-*STK11*^shRNA^ exhibited a diffuse cytoplasmic green fluorescence upon HNK treatment (Fig. 5b, c). Rab7 is a small GTPase that gets activated and recruited to early endosomes to mediate vesicle tethering and fusion of autophagosomes and lysosomes via interactions with other effector proteins^37,38^. MCF7-vector and MCF7-STK11^shRNA^ cells were co-transfected with GFP-LC3B and RFP-Rab7 plasmids to examine the formation of autophagosomes (GFP-positive/RFP-negative, green puncta), lysosomes (GFP-negative/RFP-positive, red puncta) or autophagosome-lysosome fusion (GFP-positive/RFP-positive, yellow puncta) upon HNK treatment. MCF7-STK11^shRNA^ cells showed a diffuse staining for green and red fluorescence while MCF7-vector cells exhibited increased fusion of autophagosomes-lysosomes. MCF7-STK11^shRNA^ cells showed higher RFP-Rab7 signal in untreated cells which was not observed upon HNK treatment (Fig. 5d, g). Furthermore, we examined the involvement of STK11 in the lysosomal proteolytic activity necessary to degrade cargo in the autolysosomes. This key step in autophagic process can be assayed by using DQ-BSA (a derivative of BSA whose green fluorescence is quenched except when it is cleaved by proteolytic enzymes) assay. MCF7-vector cells presented a stimulated lysosomal activity as evident by dequenching of DQ-BSA in lysosomes (elevated green signal) and an overlap with LysoTracker red signal (merged as yellow) in response to HNK treatment. In contrast, HNK-treated MCF7-STK11^shRNA^ cells demonstrated a reduction in lysosomal activity as evident by quenching of DQ-BSA signal and a diffuse staining with LysoTracker red (Fig. 5e, f). Together, these results confirm that breast cancer cells undergo autophagy in a STK11-dependent manner.

### Concomitant treatment with autophagy inhibitor and HNK synergistically inhibits breast cancer

**Fig. 6:**
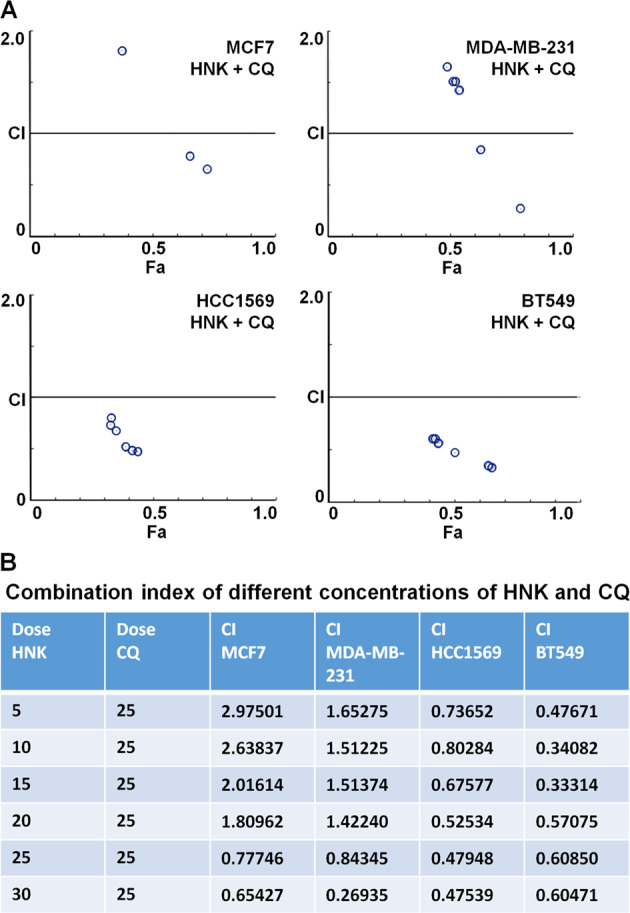
Combined treatment with HNK and CQ synergistically inhibits breast cancer cells. **a** MCF7, MDA-MB-231, HCC1569, and BT549 breast cancer cells were treated with various concentration of HNK (5.0, 10.0, 15.0, 20.0, 25.0, and 30.0µM) in combination with 25µM of CQ for 24h. Cells were subjected to MTT assay and combination index values were calculated using CompuSyn software. CI < 1 shows synergism, CI = 1 shows additivity and CI > 1 shows antagonism. **b** Table shows combination index for different concentrations of HNK and CQ.

**Fig. 7:**
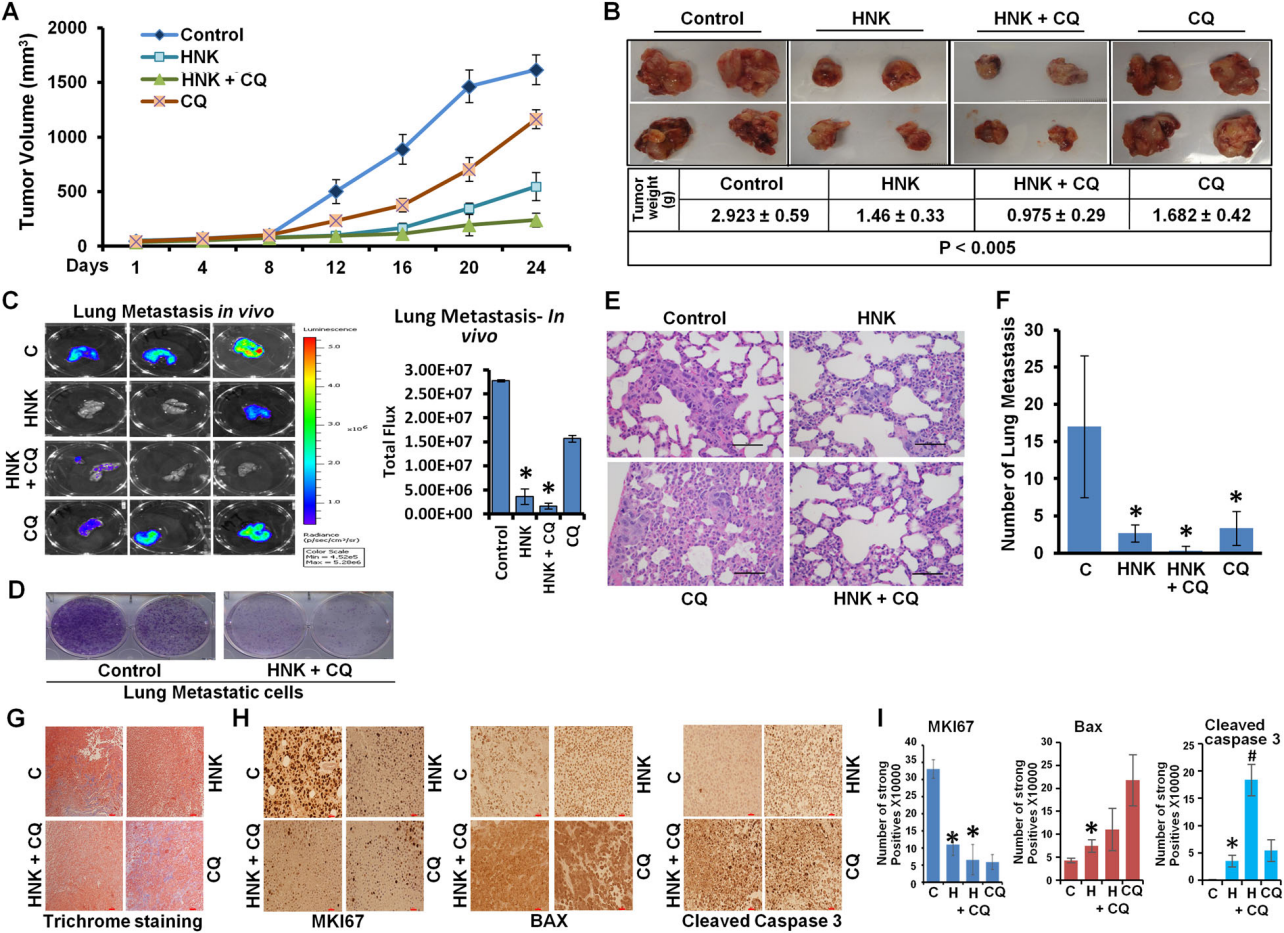
Combined HNK+CQ treatment inhibits breast tumor growth more effectively compared to HNK alone. **a** Tumors derived from MDA-MB-231-Luc cells were developed in NOD-SCID mice and treated with control (vehicle), HNK, HNK with CQ and CQ alone. Tumor growth was monitored by measuring the tumor volume for 24 days (*n* = 5). (*P* < 0.05, HNK + CQ compared with HNK; *P* < 0.005, HNK + CQ compared with vehicle control). **b** Representative tumor images and tumor weight are shown in the table. **c** Metastatic lesions in lungs of MDA-MB-231-Luc tumor-bearing NOD-SCID mice were observed. Representative ex vivo images of lungs are shown (*n* = 3). In vivo bioluminescent signal was quantified at the end of the experiment. Quantification of radiance is shown in the graph. *P < 0.05. **d** Metastatic cells in lungs were evaluated in a growth assay and compared between control and HNK + CQ group. **e, f** Histologic analysis and quantification of the area covered by metastatic lesions in lungs of control, HNK, HNK + CQ and CQ treated mice, staining: hematoxylin and eosin. **g**–**i** MDA-MB-231-luc tumors from control, HNK, HNK + CQ and CQ treated mice were subjected to trichrome staining and immunohistochemical analysis using MKI67, BAX and cleaved caspase 3 antibodies. Bar graphs show quantitation of IHC. *P < 0.05, compared with vehicle control; ^#^*P* < 0.005, compared with HNK treated.

**Fig. 8:**
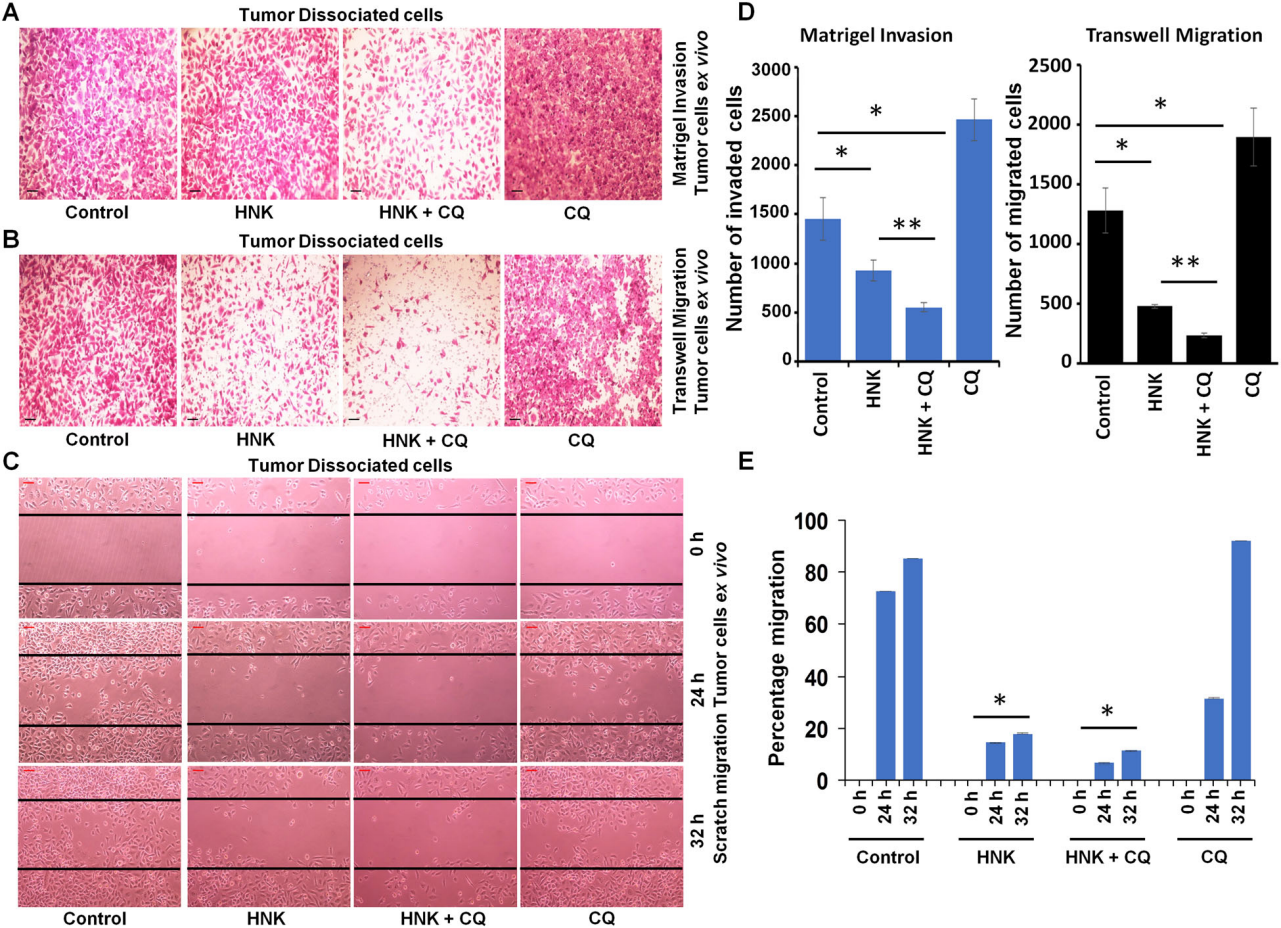
Tumor cells dissociated from primary tumors from HNK+CQ treated mice exhibit reduced migration and invasion potential. **a**–**e** Dissociated tumor cells from primary MDA-MB-231 tumors from mice treated with vehicle (control), HNK, HNK+CQ and CQ were subjected to matrigel-invasion (**a, d**), transwell-migration (**b, d**) and scratch-migration assay (**c, e**) in the absence of any additional treatment. Representative images of cells invaded or migrated are shown. Bar graphs show the average number of cells invaded/migrated and percentage migration of cells. *P < 0.05, **P < 0.005.

Our data showed that breast cancer cells undergo cytoprotective autophagy in response to HNK treatment suggesting that combining HNK with an autophagy inhibitor can potentially yield increased inhibition of breast tumor growth. Autophagy inhibitor, chloroquine has been shown to increase drug efficacy in preclinical studies^39,40^. We first determined whether the interaction between HNK and CQ is additive, synergistic or antagonistic in nature using Compusyn software (Compusyn Inc., Paramus, NJ, USA). Dose effect analyses of HNK in combination with CQ showed significant synergistic interactions in MCF7, MDA-MB-231, HCC 1569, and BT549 breast cancer cells for higher concentrations of HNK. Lower concentrations of HNK show synergistic effects in HCC 1569 and BT549 cells (Fig. 6a, b). Subsequently, the in vivo physiological relevance of our in vitro results was investigated by evaluating whether oral administration of HNK and CQ combination yields improved breast tumor inhibition in comparison to HNK alone. Xenografts of MDA-MB-231-luc cells were developed in NOD-SCID mice followed by treatment with vehicle, HNK, CQ or HNK + CQ combination for ~4 weeks. Breast tumor growth was inhibited with HNK alone treatment but the combined HNK and CQ treatment indeed achieved greater tumor inhibition (Fig. 7a). Tumor burden was significantly reduced in mice treated with HNK + CQ combination in comparison to single HNK treatment (Fig. 7b). Importantly, HNK + CQ treatment significantly reduced metastatic lesions in lungs as observed in *ex vivo* bioluminescent imaging of lungs (Fig. 7c). Metastatic cells from lungs of mice treated with vehicle or HNK + CQ combination were evaluated in a clonogenicity assay and decreased clonogenic potential was observed in HNK + CQ group (Fig. 7d). Histopathological analyses of lungs from mice treated with vehicle, CQ, HNK, or HNK + CQ showed significantly decreased levels of metastatic lesions in mice treated with combination treatment in comparison to HNK treatment (Fig. 7e, f). Reduced level of collagen fibers were observed in breast tumors from mice treated with HNK + CQ combination in comparison to HNK-treated group as evident in trichrome staining (Fig. 7g). Further analysis of breast tumors showed reduced levels of MKI67 and elevated levels of Bax and cleaved caspase 3 in HNK group in comparison to vehicle-treated group while HNK + CQ group exhibited lowest expression of MKI67 and highest expression of Bax and cleaved caspase 3 (Fig. 7h, i). Tumor-dissociated cells from breast tumors from all treatment groups were examined for migration and invasion potential. Interestingly, tumor-dissociated cells from HNK + CQ group demonstrated lowest invasion and migration potential (Fig. 8a–e). Collectively, the in vitro and in vivo findings presented here reveal that breast cancer cells initiate a cytoprotective autophagic response in a STK11-dependent manner to evade HNK efficacy which can be potentiated by combining an autophagy inhibitor with HNK treatment. Combination treatment not only inhibits breast tumor growth but also abrogates lung metastases.

## Discussion

Induction of autophagy, a cellular degradation process, helps normal cells to swiftly adapt to challenging conditions such as nutrient and growth factor deprivation, hypoxia, endoplasmic reticulum stress, and infection.

Functional impact of autophagy is very complex as it differentially affects cells based on different extracellular and intracellular signals and can be cytoprotective, cytotoxic, cytostatic, or nonprotective^27–29^. Cytotoxic or cytostatic autophagy leads to growth inhibition and apoptotic induction to promote cell death. Previous studies investigating the effect of combination regimens including vitamin D and radiation, eribulin and AURKA (aurora kinase A) inhibitor, and the bioactive compound C from *Celastrus paniculatus* showed the involvement of cytotoxic autophagy aiding apoptotic induction^41–44^. Adiponectin, an adipocytokine with anti-cancer potential, also induces cytotoxic autophagy to inhibit breast tumor progression^45^. Autophagic cell death has been reported in breast cancer cells where cells undergo autophagy as a prerequisite to apoptosis either via canonical pathway involving BECN1 or noncanonical pathway independent of BECN1^28^. Interestingly, cancer cells also utilize this physiologically important process to survive the changing microenvironment during tumor growth and metastatic progression or to survive cytotoxic chemotherapy^46^. By recycling damaged cytoplasmic constituents, autophagy can help cancer cells meet their high bio-energetic demands in low-nutrient and low-oxygen states^47^. Cancer cells induce cytoprotective autophagy upon treatment with topotecan, cyclophosphamide, temozolomide, and gemcitabine to block the apoptotic pathway induced by these drugs^48–50^. In fact, drug-resistance remains the main hindrance to effective cancer therapy and many signaling pathways related to intrinsic and acquired resistance converge on the induction of cytoprotective autophagy. It is important to decipher whether cancer cells initiate autophagy in response to any cancer therapy as it can potentially impact drug efficacy either positively in case of cytotoxic autophagy or negatively in case of cytoprotective autophagy. In this study, we provide clear evidence that breast cancer cells exhibit an accumulation of autophagosomes and increased autophagosome-lysosome fusion upon HNK treatment initiating cytoprotective autophagy as inhibiting autophagy with 3-MA or Baf or knockout of *BECN1* and *ATG7* potentiates HNK-mediated growth inhibition and apoptotic induction.

Recent preclinical studies from our group and others have shown the efficacy of HNK as a potential therapy for multiple cancer types including prostate cancer, lung cancer, colon, and breast cancer^19–23,51–53^. In addition, several research groups have developed nano-HNK strategies. Hyaluronic acid-modified liposomal honokiol nanocarrier inhibit breast tumor growth and metastasis^54^. Delivery of HNK using mPEG-PLA/vitamin E-TPGS micelles via intravenous route shows lower toxicity and high efficacy^55^. Nanoencapsulation of HNK improves cisplatin-induced pathological changes via reducing cellular oxidative damage and maintaining cellular localization of mitochondrial enzyme cytochrome C^56^. Another interesting approach was to develop self-assembled microbubbles and these honokiol-loaded poly(ε-caprolactone)-poly(ethylene glycol)-poly(ε-caprolactone) (PCL-PEG-PCL, PCEC) microbubbles (HK-PCEC-MB) efficiently inhibit cisplatin-resistant ovarian cancer in vivo^57^. While preclinical studies have uncovered the underlying signaling mechanisms of HNK, developed various nano-HNK strategies, and showed anti-cancer efficacy of HNK in multiple cancer models, clinical studies are still lacking. In addition to advancement of pharmacological and toxicity studies, there is a need to develop tangible biomarkers for clinical studies to further develop this bioactive agent.

Tumor suppressor and upstream kinase, STK11, responds to various extracellular and intracellular cues and modulates cell cycle, cell polarity, cell proliferation, apoptosis, cell migration and cell metabolism by activating AMPK, AMPK-related kinases and downstream tumor suppressor proteins such as TSC1/TSC2 (TSC complex subunit 1/2)^58^. STK11 has also been reported to engage with ATG proteins and promote autophagy in cancer cells modulating the effects of anti-cancer agents^45,59,60^. Formation of a ternary complex with STRADα and MO25 promotes nuclear export and kinase activity of STK11^61^. In addition to its well-known cytoplasmic functions, STK11 also functions as a coregulator in nucleus in a p53-dependent manner^62^. STK11 can be modulated by several other kinases such as ribosomal protein S6 kinase A1 (RPS6KA1), mitogen-activated protein kinase 1 (MAPK), AKT1, and aurora kinase A (AURKA); posttranslational modifications including farnesylation, acetylation, ubiquitination, SUMOylation; and bioactive compound HNK^23,58^. Recent study from our group reported that STK11 plays an important role in mediating anti-cancer effects of HNK leading to inhibition of cancer stem-like phenotype in breast cancer by direct inhibition of oncogenic Stat3 signaling^23^. HNK upregulates expression of STK11 and higher expression of STK11 positively correlates with breast cancer prognosis^23^. The present study shows the complexity of this pathway as HNK-mediated upregulation of STK11 also supports a negative feedback loop triggering a cytoprotective autophagy in addition to stimulating breast cancer cell death. These findings suggest that inhibition of STK11 will likely compromise the overall efficacy of HNK as it will also abrogate HNK-STK11-growth inhibition axis whereas sole inhibition of cytoprotective autophagy can potentiate the anti-cancer efficacy of HNK. Indeed, a combined strategy of treating breast tumors with HNK and CQ leads to greater inhibition of tumor growth and metastasis.

Chemical or molecular inhibition of autophagy in breast cancer stem cells reverses chemoresistance in triple negative breast cancer^63^. Combining chloroquine with isorhamnetin, a flavonoid^64^, curcumin^65^, chemotherapy (adriamycin + cyclophosphamide)^66^, and docetaxel-loaded dendritic copolymer nanoparticles^67^ improves the therapeutic potential of these agents. Efficacy of autophagy inhibition using FDA-approved chloroquine or hydroxychloroquine in combination with chemotherapy is being investigated in many clinical trials^68–72^. While few FDA-approved autophagy inducers such as chloroquine, hydroxychloroquine, rapamycin and its analogs are being investigated in clinical studies, several new autophagy inhibitors are also being developed and tested namely, 2-amino-nicotinonitrile compound (w09)^73^, 5-amino-2-ether-benzamide derivatives^74^, STF-62247, and pimozide^75^. Future studies may show efficacy of these novel autophagy inhibitors in combination with standard anti-cancer therapeutic regimens. In conclusion, we present that breast cancer cells invoke cytoprotective autophagy in response to HNK treatment via STK11 whose expression is a key node for autophagic induction. Overcoming cytoprotective autophagy by combining HNK and CQ potentiates the efficacy of HNK resulting in greater inhibition of breast tumor growth and metastatic progression.

## Materials and methods

### Cell culture and reagents

Human breast cancer cell lines MCF7, MDA-MB-231, HCC1569, BT549, and MDA-MB-468 were purchased from American Type Culture Collection (ATCC), and SUM149 and SUM159 were procured from Asterand Bioscience (Detroit, MI). Cells were thawed from early passage liquid nitrogen vapor stocks as needed and cultured according to the supplier’s instruction. Honokiol (HNK) is a natural product extracted from the seed cone of *Magnolia grandiflora* as previously described^76^. Previous studies from our lab showed that 5 µM HNK is the effective dose for in vitro studies and 150 mg/kg, oral gavage, thrice a week HNK is effective for in vivo studies for breast cancer cells and tumor models, respectively^19,21–23^. Antibodies for MAP1LC3B/LC3B (3868), ATG5 (12994), ATG7 (8558), BECN1 (3495), STK11 (3050), pATG1, cleaved PARP1 (5625), PARP1 (9532), pAMPK, AMPK, p4EBP1, and pS6K were obtained from Cell Signaling Technology. ACTB/β-actin (A5441) antibody, 3-Methyladenine (M9281), Chloroquine (C6628) and Rapamycin (R0395) were purchased from Sigma-Aldrich. Bafilomycin A1 (BAI-1) was purchased from Cayman Chemical. DQ^TM^ Green BSA assay (D12050), Earle’s Balanced Salt Solution (EBSS; 14155-063), Alexa Fluor 488 (A-11008) and Alexa Fluor 555 (A-21428) were purchased from Thermo Fisher Scientific. LysoTracker Red DND-99 (L7528) was purchased from Invitrogen. Plasmids for EGFP-LC3B and mRFP-EGFP-tfLC3B were purchased from Addgene (Cambridge, MA).

### TEM, immunofluorescence, and confocal imaging

Breast cancer cells were treated with HNK for 24 h, and fixed with electron microscopy fixing buffer, rinsed, stained with 2% uranyl acetate (0.22 µm filtered, 1 h, dark) in 0.1 M maleate buffer and dehydrated through a graded series of ethanol (30–100%). Cells were embedded in EPON, sectioned, stained and examined with an H7600 transmission electron microscope (Hitachi, Tokyo, Japan)^45^. Breast cancer cells (5 ×10^5^ cells/well) were plated in 4-well chamber slides (Nunc, Rochester, NY) followed by HNK treatment as indicated and subjected to immunofluorescence analysis^77^. Fixed and immunofluorescently stained cells were imaged using a Zeiss LSM510 Meta (Zeiss, Dublin, California, USA) laser scanning confocal system configured to a Zeiss Axioplan 2 upright microscope (Zeiss, Dublin, California, USA).

### Acridine orange staining, LysoTracker Red staining, mRFP-EGFP-LC3B assay, and transfection

For *acridine orange staining*, breast cancer cells were cultured on chamber slides, treated with HNK as indicated followed by incubation with acridine orange (Sigma-Aldrich, A9231) and imaged using confocal microscope. *LysoTracker Red staining* was used to measure the intralysosomal function. Briefly, breast cancer cells were transfected with GFP-LC3B construct using FuGene HD transfection reagent (Promega), treated with HNK as indicated and incubated with 50 nM LysoTracker Red DND-99, and the fluorescence intensity was observed under the confocal microscope. For *DQ-BSA assay*, breast cancer cells were incubated with 10 µg/ml DQ-BSA for 2 h followed by treatment with 5 µM HNK. Cells were fixed and stained with LysoTracker Red followed by immunofluoroscent imaging. For *co-staining with Rab7 and LC3B*, breast cancer cells were transfected with GFP-LC3B and RFP-Rab7 using FuGene HD transfection reagent (Promega), treated with HNK as indicated and examined under the confocal microscope. For the *mRFP-EGFP-LC3B assay*, breast cancer cells were seeded 4-well chamber slides, transfected with mRFP-EGFP-LC3B (Addgene, 21074; deposited by Tamotsu Yoshimori) using FuGene HD transfection reagent (Promega), treated with HNK as indicated, and examined under the confocal microscope. Multiple view-fields were examined and representative cells from 3 view-fields were photographed. For puncta, at least 50–100 cells/sample were counted in triplicates^45,77,78^.

### Immunoblot analysis

The whole cell lysate was prepared by scraping cells in 100 μl of ice-cold modified RIPA buffer^79^, total protein quantified using Bradford protein assay kit (Bio-Rad, Hercules, CA), equal amount of proteins was resolved on sodium-dodecyl sulfate polyacrylamide gel (SDS-PAGE), transferred to nitrocellulose membrane and western blot analysis was performed. Immunodetection was performed using enhanced chemiluminescence (ECL system, Amersham Pharmacia Biotech Inc., Arlington Heights, IL) according to manufacturer’s instructions.

### STK11 stable knockdown using lentiviral short-hairpin RNA

Five pre-made lentiviral STK11 short-hairpin RNA (shRNA) constructs and a negative control construct created in the same vector system (pLKO.1) were purchased from Open Biosystems (Huntsville, AL). Paired STK11 stable knockdown cells was generated by following our previously published protocol^45,80^.

### ATG7 and BECN1 knockout with CRISPR/Cas9

For *ATG7* and *BECN1* knockout, we digested and purified LentiCRISPRv2 plasmid [lentiCRISPRv2, a gift from Feng Zhang (Addgene, 52961)], incubated with phosphorylated, annealed oligos for *ATG7* and *BECN1* in a ligation reaction, transformed into Stbl3 bacteria (ThermoFisher Scientific, C7373-03). lentiCRISPR with inserted sequences were co-transfected into HEK293T cells with packinging plasmids. MCF7 cells were transfected twice, and selected for a week. MCF7 cells were examined for the *ATG7* and *BECN1* knockout using immunoblot analyses^45,77^.

### Cell viability, clonogenicity assay, and apoptosis

The *MTT assay* was performed by estimating the reduction of MTT (3-(4, 5-dimethylthiazol-2-yl)-2, 5-diphenyltetrazolium bromide). Cells were plated in 96 well plates at an initial density of 5 ×10^3^ cells per well for 24 h. After 24 h, the culture medium was changed to medium containing treatment as indicated. MTT reagent was added to each culture well to attain a final concentration of 0.5 mg/ml and incubated for 4 h at 37 °C. The quantity of formazan (presumably directly proportional to the number of viable cells) was dissolved using solubilization solution (10% SDS) and incubated for 8–12 h at 37 °C, measured by absorbance at 570 nm using a 96 well plate reader. *Cell viability* was assessed by trypan blue exclusion assay. Breast cancer cells were harvested using trypsin (0.2%), stained with trypan blue (Sigma) and counted using a hemocytometer under the phase contrast microscopy. For *clonogenicity assay*, cells were treated, counted and plated in 6-well plates at a density of 1 ×10^3^ cells per well and incubated for a week. Post-incubation, colonies were washed with phosphate buffered saline (PBS), fixed with formalin, stained with 0.1% crystal violet (Sigma) and air-dried. The pictures were taken under the phase contrast microscopy. For *apoptosis assay*, cells were cultured on chamber slides, treated as described followed by Hoechst staining. Hoechst-positive (apoptotic) cells were counted in treatment and control (cells grown in complete medium containing 10% fetal bovine serum). Data from 10 fields were collected for each treatment condition. *DNA-Fragmentation Assay* was conducted using DNA Fragmentation Imaging Kit (Sigma-Aldrich) following manufacturer’s instructions.

### Breast tumorigenesis assay

MDA-MB-231-Luc cells were implanted in mammary glands of 7–8 weeks old female NOD-SCID mice. MDA-MB-231-luc cells metastasize to lungs. Tumor-bearing mice were randomly grouped in four experimental groups and oral-gavaged with vehicle (intralipid), HNK (3 mg/mice/thrice a week), chloroquine (1 μg/mice/day) and HNK + chloroquine for 4 weeks. Tumor progression was measured regularly. At the end of the experiment, ex vivo bioluminescent images of lungs were captured to investigate metastatic progression. Briefly, animals were given an intraperitoneal injection of D-luciferin and were euthanized after 10 min. Lungs were excised and images were captured using IVIS system. Tumors were resected, measured, weighed, and processed for immunohistochemistry. A portion of tumors was processed for attaining tumor-dissociated cells. Tumor-dissociated cells were subjected to matrigel-invasion, scratch-migration and transwell-migration assays. For IHC, at least four random, non-overlapping representative images from each tumor section from all the tumors of each group were captured using ImagePro software for quantification. For lung metastasis assay, lungs excised from tumor-bearing mice were harvested in DMEM F12 medium. With curved scissors, lungs were minced into pieces and transferred into 15 ml tubes containing 2.5 ml of respective digestion cocktail (RPMI + 10 mg/ml of Collagenase A + 10 mg/ml of Hylauronidase for lungs. The organs were then placed in shaking water bath at 37 °C for 30 min to allow complete dissociation. After enzymatic digestion, volumes of the samples were made up to 10 ml with PBS and each sample was filtered through separate 70 μm nylon cell strainer to remove large chunks of undigested tissue. Samples were collected in 50 ml tubes, centrifuged for 5 min at 1500 rpm, RT, in a bench top centrifuge and supernatant was discarded. Samples were washed twice by centrifugation in PBS. Pellets were resuspended in culture media and plated onto 6-well culture plates. Plates were incubated in 37 °C tissue culture incubator, 5% CO_2_ to allow growth of colonies for 3–7 days. All animal studies were in accordance with the guidelines of Johns Hopkins University IACUC.

### Statistical analysis

All experiments were performed thrice in triplicates. Statistical analysis was performed using Microsoft Excel software. Significant differences were analyzed using the Student’s *t* test and two-tailed distribution. Results were considered to be statistically significant if *P* < 0.05. Results were expressed as mean ± SE between triplicate experiments performed thrice.

## Supplementary information

Legends to Supplementary Figures

Supplementary Figure 1

Supplementary Figure 2

Supplementary Figure 3

## References

[1] Henley, S. J. et al. Annual Report to the Nation on the Status of Cancer, part I: National cancer statistics. *Cancer*, 10.1002/cncr.32802 (2020). 10.1002/cncr.32802 PMC7299151 32162336

[2] Henley, S. J. et al. Annual Report to the Nation on the Status of Cancer, part II: Progress toward Healthy People 2020 objectives for 4 common cancers. *Cancer*, 10.1002/cncr.32801 (2020). 10.1002/cncr.32801 PMC7223723 32162329

[3] Lozy F, Karantza V (2012). Autophagy and cancer cell metabolism. Semin. Cell Dev. Biol..

[4] Klionsky DJ (2005). The molecular machinery of autophagy: unanswered questions. J. Cell Sci..

[5] Mizushima N, Komatsu M (2011). Autophagy: renovation of cells and tissues. Cell.

[6] Kroemer G, Marino G, Levine B (2010). Autophagy and the integrated stress response. Mol. Cell.

[7] Yorimitsu T, Klionsky DJ (2005). Autophagy: molecular machinery for self-eating. Cell Death Differ..

[8] Klionsky DJ (2012). A human autophagy interaction network. Autophagy.

[9] Yu L (2010). Termination of autophagy and reformation of lysosomes regulated by mTOR. Nature.

[10] Song J (2009). Hypoxia-induced autophagy contributes to the chemoresistance of hepatocellular carcinoma cells. Autophagy.

[11] Yoon JH, Ahn SG, Lee BH, Jung SH, Oh SH (2012). Role of autophagy in chemoresistance: regulation of the ATM-mediated DNA-damage signaling pathway through activation of DNA-PKcs and PARP-1. Biochem. Pharm..

[12] Galluzzi L (2014). Systems biology of cisplatin resistance: past, present and future. Cell Death Dis..

[13] Fridlender M, Kapulnik Y, Koltai H (2015). Plant derived substances with anti-cancer activity: from folklore to practice. Front Plant Sci..

[14] Fried LE, Arbiser JL (2009). Honokiol, a multifunctional antiangiogenic and antitumor agent. Antioxid. Redox Signal.

[15] Wolf I (2007). Honokiol, a natural biphenyl, inhibits in vitro and in vivo growth of breast cancer through induction of apoptosis and cell cycle arrest. Int. J. Oncol..

[16] Liu H (2008). Anti-tumor effect of honokiol alone and in combination with other anti-cancer agents in breast cancer. Eur. J. Pharm..

[17] Park EJ (2009). Down-regulation of c-Src/EGFR-mediated signaling activation is involved in the honokiol-induced cell cycle arrest and apoptosis in MDA-MB-231 human breast cancer cells. Cancer Lett..

[18] Singh T, Katiyar SK (2011). Honokiol, a phytochemical from Magnolia spp., inhibits breast cancer cell migration by targeting nitric oxide and cyclooxygenase-2. Int. J. Oncol..

[19] Nagalingam A, Arbiser JL, Bonner MY, Saxena NK, Sharma D (2012). Honokiol activates AMP-activated protein kinase in breast cancer cells via an LKB1-dependent pathway and inhibits breast carcinogenesis. Breast Cancer Res..

[20] Avtanski DB (2014). Honokiol inhibits epithelial-mesenchymal transition in breast cancer cells by targeting signal transducer and activator of transcription 3/Zeb1/E-cadherin axis. Mol. Oncol..

[21] Avtanski DB (2015). Honokiol abrogates leptin-induced tumor progression by inhibiting Wnt1-MTA1-beta-catenin signaling axis in a microRNA-34a dependent manner. Oncotarget.

[22] Avtanski DB (2015). Honokiol activates LKB1-miR-34a axis and antagonizes the oncogenic actions of leptin in breast cancer. Oncotarget.

[23] Sengupta S (2017). Activation of tumor suppressor LKB1 by honokiol abrogates cancer stem-like phenotype in breast cancer via inhibition of oncogenic Stat3. Oncogene.

[24] Green DR, Levine B (2014). To be or not to be? How selective autophagy and cell death govern cell fate. Cell.

[25] Mizushima N, Yoshimori T, Levine B (2010). Methods in mammalian autophagy research. Cell.

[26] Kimura S, Noda T, Yoshimori T (2007). Dissection of the autophagosome maturation process by a novel reporter protein, tandem fluorescent-tagged LC3. Autophagy.

[27] Gewirtz DA (2014). The four faces of autophagy: implications for cancer therapy. Cancer Res..

[28] Sharma K, Le N, Alotaibi M, Gewirtz DA (2014). Cytotoxic autophagy in cancer therapy. Int. J. Mol. Sci..

[29] Gewirtz DA (2014). When cytoprotective autophagy isn’t… and even when it is. Autophagy.

[30] Wu YT (2010). Dual role of 3-methyladenine in modulation of autophagy via different temporal patterns of inhibition on class I and III phosphoinositide 3-kinase. J. Biol. Chem..

[31] Rubinsztein DC (2009). In search of an “autophagomometer”. Autophagy.

[32] Nagelkerke A, Sweep FC, Geurts-Moespot A, Bussink J, Span PN (2015). Therapeutic targeting of autophagy in cancer. Part I: molecular pathways controlling autophagy. Semin. Cancer Biol..

[33] Vaahtomeri K, Makela TP (2011). Molecular mechanisms of tumor suppression by LKB1. FEBS Lett..

[34] Hardie DG (2005). New roles for the LKB1–>AMPK pathway. Curr. Opin. cell Biol..

[35] Lu C, Xie C (2016). Radiation-induced autophagy promotes esophageal squamous cell carcinoma cell survival via the LKB1 pathway. Oncol. Rep..

[36] Sun A (2016). GSK-3beta controls autophagy by modulating LKB1-AMPK pathway in prostate cancer cells. Prostate.

[37] Rink J, Ghigo E, Kalaidzidis Y, Zerial M (2005). Rab conversion as a mechanism of progression from early to late endosomes. Cell.

[38] Markgraf DF, Peplowska K, Ungermann C (2007). Rab cascades and tethering factors in the endomembrane system. FEBS Lett..

[39] Fleisher B, Mody H, Werkman C, Ait-Oudhia S (2019). Chloroquine sensitizes MDA-MB-231 cells to osimertinib through autophagy-apoptosis crosstalk pathway. Breast Cancer (Dove Med Press).

[40] Gao L (2018). Histone deacetylase inhibitor trichostatin A and autophagy inhibitor chloroquine synergistically exert anti-tumor activity in H-ras transformed breast epithelial cells. Mol. Med. Rep..

[41] Bristol ML (2012). Dual functions of autophagy in the response of breast tumor cells to radiation: cytoprotective autophagy with radiation alone and cytotoxic autophagy in radiosensitization by vitamin D 3. Autophagy.

[42] Wilson EN (2011). A switch between cytoprotective and cytotoxic autophagy in the radiosensitization of breast tumor cells by chloroquine and vitamin D. Hormones Cancer.

[43] Kozyreva VK (2016). Combination of eribulin and aurora A inhibitor MLN8237 Prevents metastatic colonization and induces cytotoxic autophagy in breast cancer. Mol. Cancer Therapeutics.

[44] Weng JR, Yen MH, Lin WY (2013). Cytotoxic constituents from Celastrus paniculatus induce apoptosis and autophagy in breast cancer cells. Phytochemistry.

[45] Chung SJ (2017). ADIPOQ/adiponectin induces cytotoxic autophagy in breast cancer cells through STK11/LKB1-mediated activation of the AMPK-ULK1 axis. Autophagy.

[46] Buchser WJ, Laskow TC, Pavlik PJ, Lin HM, Lotze MT (2012). Cell-mediated autophagy promotes cancer cell survival. Cancer Res..

[47] Mathew R, Karantza-Wadsworth V, White E (2007). Role of autophagy in cancer. Nat. Rev. Cancer.

[48] Li DD (2009). The pivotal role of c-Jun NH2-terminal kinase-mediated Beclin 1 expression during anticancer agents-induced autophagy in cancer cells. Oncogene.

[49] Oehadian A (2007). Differential expression of autophagy in Hodgkin lymphoma cells treated with various anti-cancer drugs. Acta Med. Indones..

[50] Kanzawa T (2004). Role of autophagy in temozolomide-induced cytotoxicity for malignant glioma cells. Cell Death Differ..

[51] Wynn ML, Consul N, Merajver SD, Schnell S (2014). Inferring the effects of honokiol on the notch signaling pathway in SW480 colon cancer cells. Cancer Inf..

[52] Crane C, Panner A, Pieper RO, Arbiser J, Parsa AT (2009). Honokiol-mediated inhibition of PI3K/mTOR pathway: a potential strategy to overcome immunoresistance in glioma, breast, and prostate carcinoma without impacting T cell function. J. Immunother..

[53] Zhu J, Xu S, Gao W, Feng J, Zhao G (2019). Honokiol induces endoplasmic reticulum stress-mediated apoptosis in human lung cancer cells. Life Sci..

[54] Wang J (2020). Hyaluronic acid-modified liposomal honokiol nanocarrier: enhance anti-metastasis and antitumor efficacy against breast cancer. Carbohydr. Polym..

[55] Sun J (2020). Tuning mPEG-PLA/vitamin E-TPGS-based mixed micelles for combined celecoxib/honokiol therapy for breast cancer. Eur. J. Pharm. Sci..

[56] Liu HT (2019). Nanoparticulated honokiol mitigates cisplatin-induced chronic kidney injury by maintaining mitochondria antioxidant capacity and reducing Caspase 3-associated cellular apoptosis. Antioxidants (Basel).

[57] Wu Q, Zhang M, Luo H, Yi T (2018). Self-assembled honokiol-loaded microbubbles in the treatment of ovarian cancer by ultrasound irradiation. J. Biomed. Nanotechnol..

[58] Kullmann L, Krahn MP (2018). Controlling the master-upstream regulation of the tumor suppressor LKB1. Oncogene.

[59] Jin P (2019). MCT1 relieves osimertinib-induced CRC suppression by promoting autophagy through the LKB1/AMPK signaling. Cell Death Dis..

[60] Li F (2019). BET inhibitor JQ1 suppresses cell proliferation via inducing autophagy and activating LKB1/AMPK in bladder cancer cells. Cancer Med..

[61] Woods A (2003). LKB1 is the upstream kinase in the AMP-activated protein kinase cascade. Curr. Biol..

[62] Zeng PY, Berger SL (2006). LKB1 is recruited to the p21/WAF1 promoter by p53 to mediate transcriptional activation. Cancer Res..

[63] Bousquet G (2017). Targeting autophagic cancer stem-cells to reverse chemoresistance in human triple negative breast cancer. Oncotarget.

[64] Hu J (2019). ROS-mediated activation and mitochondrial translocation of CaMKII contributes to Drp1-dependent mitochondrial fission and apoptosis in triple-negative breast cancer cells by isorhamnetin and chloroquine. J. Exp. Clin. Cancer Res..

[65] Masuelli L (2017). Chloroquine supplementation increases the cytotoxic effect of curcumin against Her2/neu overexpressing breast cancer cells in vitro and in vivo in nude mice while counteracts it in immune competent mice. Oncoimmunology.

[66] Lefort S (2014). Inhibition of autophagy as a new means of improving chemotherapy efficiency in high-LC3B triple-negative breast cancers. Autophagy.

[67] Zhang X (2014). Enhancing therapeutic effects of docetaxel-loaded dendritic copolymer nanoparticles by co-treatment with autophagy inhibitor on breast cancer. Theranostics.

[68] Mahalingam D (2014). Combined autophagy and HDAC inhibition: a phase I safety, tolerability, pharmacokinetic, and pharmacodynamic analysis of hydroxychloroquine in combination with the HDAC inhibitor vorinostat in patients with advanced solid tumors. Autophagy.

[69] Rangwala R (2014). Combined MTOR and autophagy inhibition: phase I trial of hydroxychloroquine and temsirolimus in patients with advanced solid tumors and melanoma. Autophagy.

[70] Vogl DT (2014). Combined autophagy and proteasome inhibition: a phase 1 trial of hydroxychloroquine and bortezomib in patients with relapsed/refractory myeloma. Autophagy.

[71] Guo W, Wang Y, Wang Z, Wang YP, Zheng H (2016). Inhibiting autophagy increases epirubicin’s cytotoxicity in breast cancer cells. Cancer Sci..

[72] Sun R (2016). Nanoparticle-facilitated autophagy inhibition promotes the efficacy of chemotherapeutics against breast cancer stem cells. Biomaterials.

[73] Zhang P (2017). w09, a novel autophagy enhancer, induces autophagy-dependent cell apoptosis via activation of the EGFR-mediated RAS-RAF1-MAP2K-MAPK1/3 pathway. Autophagy.

[74] Shan, C. et al. Discovery of novel autophagy inhibitors and their sensitization abilities for vincristine-resistant esophageal cancer cell line Eca109/VCR. *ChemMedChem*10.1002/cmdc.202000004 (2020). 10.1002/cmdc.202000004 32207878

[75] Kinzler MN (2020). STF-62247 and pimozide induce autophagy and autophagic cell death in mouse embryonic fibroblasts. Sci. Rep..

[76] Bai X (2003). Honokiol, a small molecular weight natural product, inhibits angiogenesis in vitro and tumor growth in vivo. J. Biol. Chem..

[77] Muniraj, N. et al. Withaferin A inhibits lysosomal activity to block autophagic flux and induces apoptosis via energetic impairment in breast cancer cells. *Carcinogenesis*, 10.1093/carcin/bgz015 (2019). 10.1093/carcin/bgz015 PMC10893887 30698683

[78] Siddharth S, Muniraj N, Saxena NK, Sharma D (2019). Concomitant inhibition of cytoprotective autophagy augments the efficacy of withaferin a in hepatocellular carcinoma. Cancers.

[79] Yan D, Avtanski D, Saxena NK, Sharma D (2012). Leptin-induced epithelial-mesenchymal transition in breast cancer cells requires beta-catenin activation via Akt/GSK3- and MTA1/Wnt1 protein-dependent pathways. J. Biol. Chem..

[80] Taliaferro-Smith L (2009). LKB1 is required for adiponectin-mediated modulation of AMPK-S6K axis and inhibition of migration and invasion of breast cancer cells. Oncogene.

