# Supplementary material for: Induction of STK11-dependent cytoprotective autophagy in breast cancer cells upon honokiol treatment
Source: Cell Death Discov. 2020 Sep 6;6:81. doi: 10.1038/s41420-020-00315-w (PMC7475061; doi:10.1038/s41420-020-00315-w)
Supplement: Supplementary file 1 — Legends to Supplementary Figures [file 41420_2020_315_MOESM1_ESM.doc]

**Legends to Supplementary Figures**

**Supplementary Figure 1 HNK increases the expression of ATG proteins.** Immunoblot analysis of pATG1, ATG5, ATG7 and BECN1 in breast cancer cells treated with 5 µM HNK for indicated time intervals. ACTB was used as a loading control. The bar graphs represent densitometric analyses of immunoblot signals using ImageJ software.

**Supplementary Figure 2 Inhibition of autophagy increases HNK-mediated reduction in cell survival.** MCF7 **(A)** and MDA-MB-231 **(B)** cells were treated with 5 µM HNK alone or in combination with 4 mM 3-methyladenine (3MA) or 200 nM bafilomycin A_1_ (Baf) or 25 μM chloroquine (CQ) for 24 h as indicated and subjected to Trypan blue exclusion assay. * *P* < 0.05, compared with control; ^*^ *P* < 0.05, compared with HNK.

**Supplementary Figure 3 HNK increases the expression of STK11 and AMPK and decreases the expression of p-p70S6K and p-4EBP1. (A)** Total protein lysates of MCF7 cells treated with 5 µM HNK for indicated times and immunoblotted for the expression of STK11, p-AMPK and AMPK. ACTB was used as a loading control. **(B)** Total protein lysates of MCF7 and MDA-MB-231 cells treated with 5 µM HNK for 12 h and immunoblotted for the expression of p4EBP1 and p-P70S6K. ACTB was used as a loading control.
