# Supplementary figures and images for: Induction of STK11-dependent cytoprotective autophagy in breast cancer cells upon honokiol treatment
Source: Cell Death Discov. 2020 Sep 6;6:81. doi: 10.1038/s41420-020-00315-w (PMC7475061; doi:10.1038/s41420-020-00315-w)

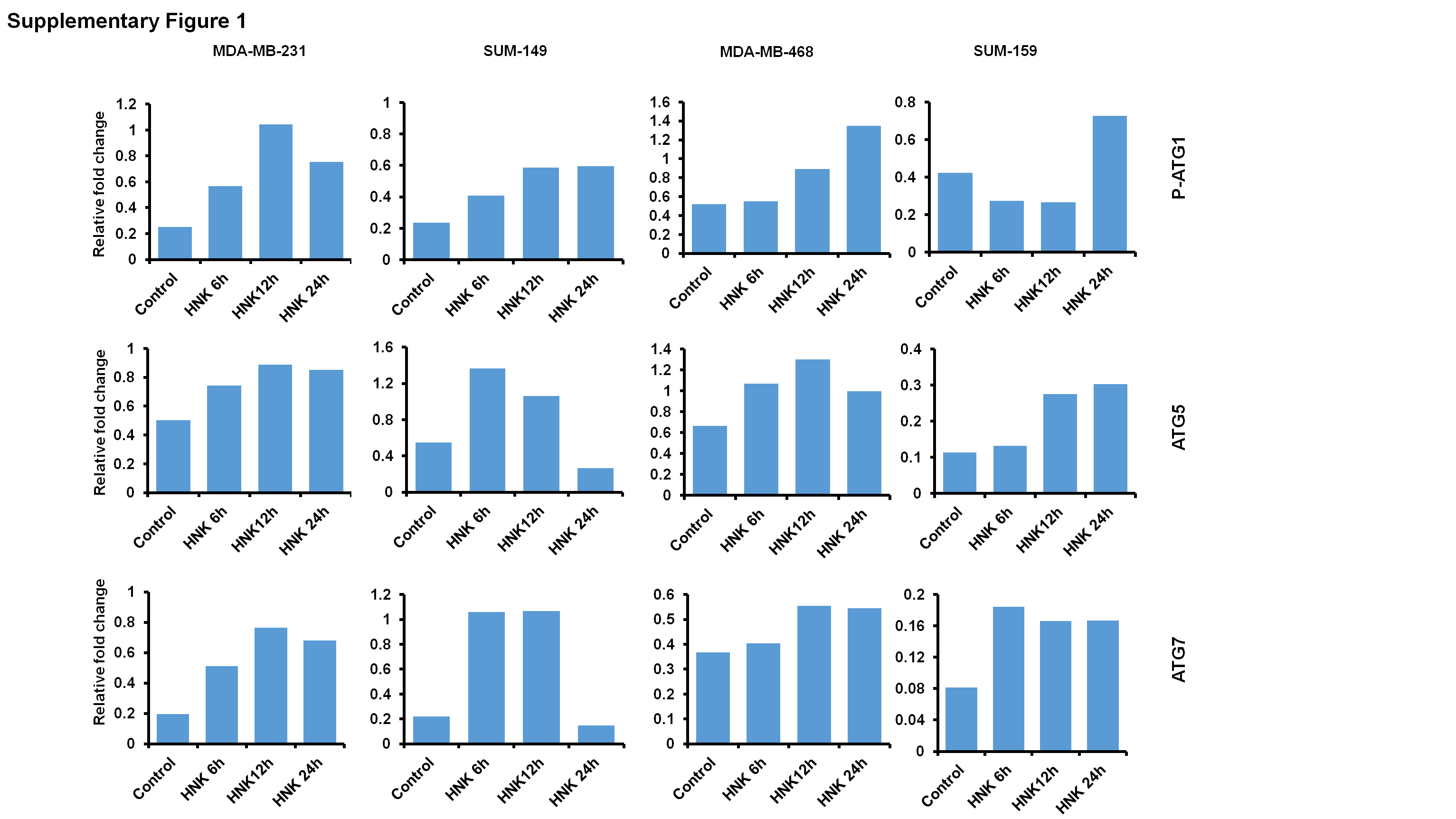

Supplement: Supplementary file 2 — Supplementary Figure 1 [file 41420_2020_315_MOESM2_ESM.tif]

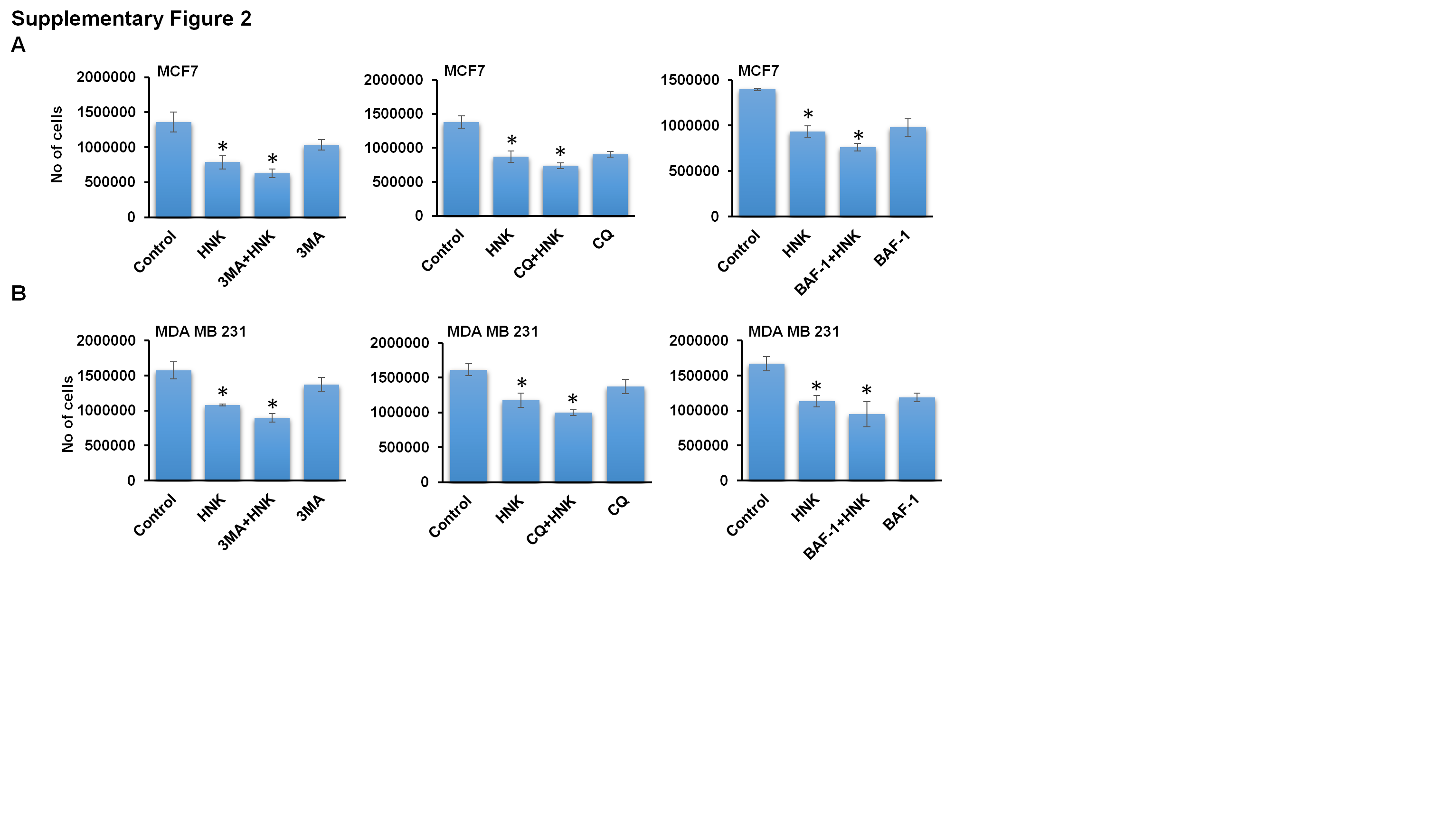

Supplement: Supplementary file 3 — Supplementary Figure 2 [file 41420_2020_315_MOESM3_ESM.tif]

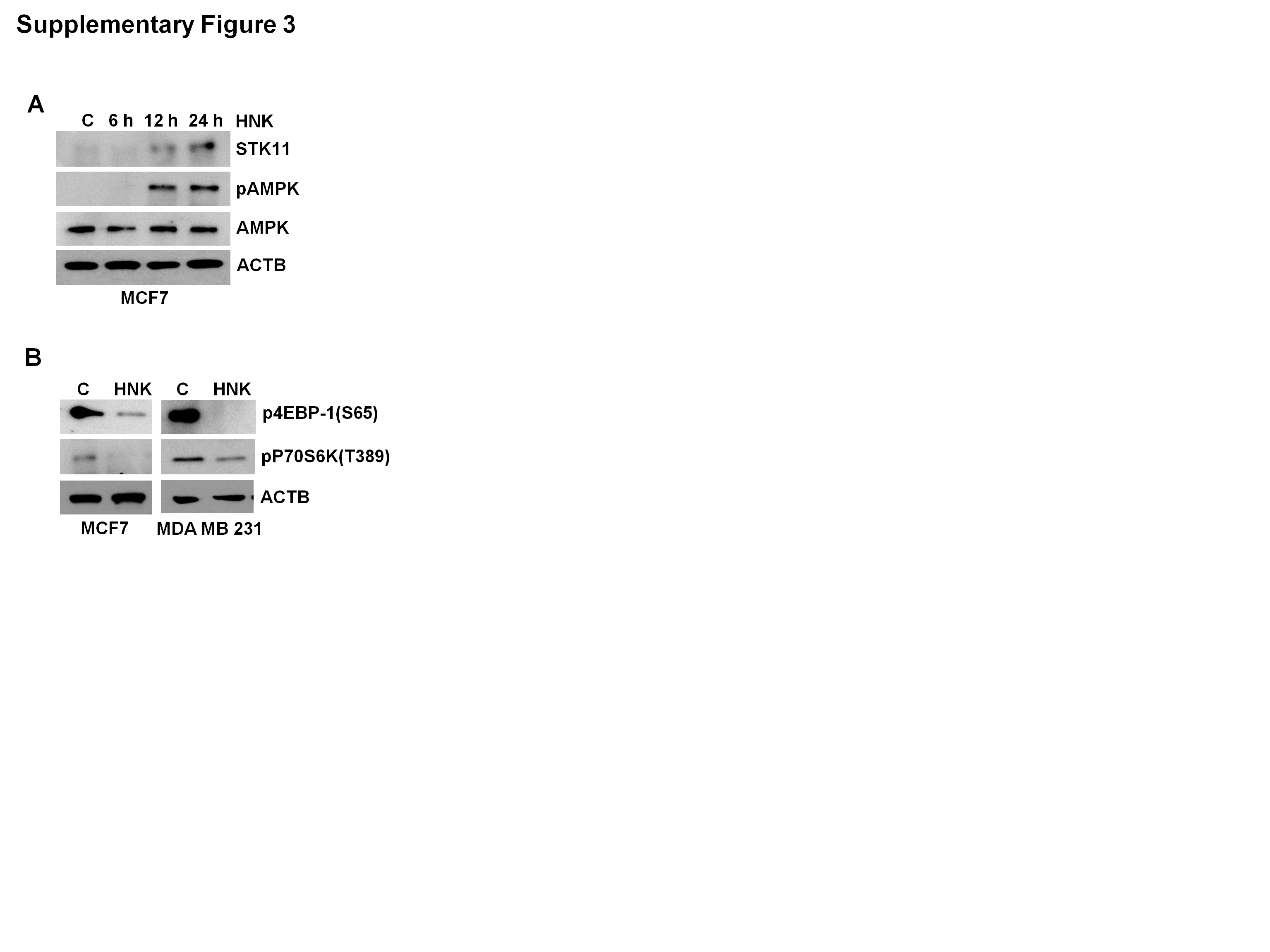

Supplement: Supplementary file 4 — Supplementary Figure 3 [file 41420_2020_315_MOESM4_ESM.tif]
